# Fungal Diversity and Potential Health Benefits of Mycophagy in Chacma Baboons (*Papio ursinus*)

**DOI:** 10.1002/ajp.70146

**Published:** 2026-04-12

**Authors:** Margaret A. H. Bryer, Carla van Hasselt, Sophie E. Kurilla, Sky B. Beveridge, Hendri C. Coetzee, Kris H. Sabbi

**Affiliations:** ^1^ Department of Anthropology University of Wisconsin‐Madison Madison Wisconsin USA; ^2^ Department of Zoology University of Glasgow Glasgow UK; ^3^ Department of Anthropology Emory University Atlanta Georgia USA; ^4^ NVT Nature's Valley South Africa; ^5^ Community Psychosocial Research Unit North‐West University Potchefstroom South Africa; ^6^ Department of Human Evolutionary Biology Harvard University Cambridge Massachusetts USA

**Keywords:** fungi, mammal‐fungal relationships, mushrooms, primate diet, primate ecology

## Abstract

Humans have consumed mushrooms for food and medicine for thousands of years, yet mycophagy remains understudied among our closest relatives, the nonhuman primates. Some species of primates feature fungi prominently in their diets, in terms of food budget or fungal diversity. Most primate species, however, are observed feeding on a general category of mushrooms for a minority of time feeding and the level of detail in reporting varies widely. In this report, we address this gap, describing fungi eaten by free‐ranging baboons (*Papio ursinus)* in Nature's Valley, South Africa. During routine behavioral observations between August 2023 and July 2025, baboons of multiple age/sex classes in Nature's Valley, South Africa, were recorded eating 13 different fungi. We identified 10 of these 13 fungi to the species level using their physical characteristics. We then assessed overlap with other reported fungal species eaten by primates as well as potential health and ecosystem implications of consumption of these fungi based on human and other mammalian mycophagy literature. Our findings suggest fungal consumption may be underestimated in some cases, but, more importantly, even when fungi are a small portion of the diet or consumed rarely, they may still play an important role that warrants deeper investigation.

## Summary

- Mycophagy, especially with fungi identified to the species level, in wild or free‐ranging nonhuman primates is poorly understood.
- We identified ten fungi to the species level that are consumed by free‐ranging chacma baboons of multiple age/sex classes.
- Some of the fungal species identified are also used as medicine by humans and have previously been analyzed for their potential as pharmaceuticals.
- Primate‐fungal relationships leave much to be explored, including in better understanding the nutrition of primate mycophagy and implications for primate and ecosystem health.

## Graphical abstract

Free‐ranging chacma baboons (*Papio ursinus*) in Nature's Valley, South Africa, of multiple age/sex classes eat diverse fungi (10 identified to species level, 3 to genus level). We assess potential nutritional, medicinal and ecosystem implications of consumption of these fungi based on human and other mammalian mycophagy literature.

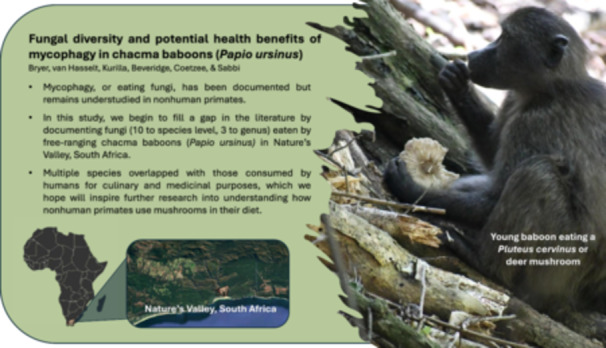

## 1 Introduction

Though humans have consumed mushrooms and other fungi for nutritional and medicinal benefits for thousands of years (Yamin‐Pasternak 2011; Roncero‐Ramos and Delgado‐Andrade 2017), the role of fungi in primate diets is underexplored. This is partially because, among primates, less feeding time is devoted to mushrooms (defined here as the observable sporing bodies, sometimes referred to as fruiting bodies, of fungi) compared to other foods (Johnson 1995; Owens et al. 2015; Takahashi et al. 2019; Watts et al. 2012; Yamagiwa et al. 2005). Further, the number of primate species for which mycophagy has been observed is relatively low (10%–20% of primate species; Hanson et al. 2003; Sawada 2014; Elliott et al. 2022), though mycophagy may be underestimated as few studies have targeted mycophagy, specifically. A second hurdle to understanding the role of fungi in primate diets is that the level of detail in available wild primate studies on how often mycophagy occurs or what fungal species are consumed varies widely in the literature (reviewed across primates by Hanson et al. 2003 and Sawada 2014; reviewed across mammals by Elliott et al. 2022). Many studies simply refer to a small percentage of the diet consisting of “mushrooms.” This variation in reporting level currently makes it challenging to understand potential roles of mushrooms and other fungi in primate diet and health. Here, we present observations of chacma baboons eating specific fungal species, identify each fungus with as much specificity as possible, and then provide potential nutritional, health, and ecosystem implications.

A few studies indicate that some primates feature fungi conspicuously in their diets, in terms of food budget or fungal diversity. Goeldi's monkeys (*Callimico goeldii*) consume the sporocarps of four fungus species, including *Auricularia auricula* (commonly known as wood ear) and *A. mesenterica* (tripe fungus) (Hanson 2000; Porter 2001; Hanson et al. 2006), devoting up to 63% of the monthly diet to mushrooms during the dry season when fruits are less available (Porter 2000). Japanese macaques (*Macaca fuscata yakui*) on Yakushima Island devote only 2% of their annual feeding time to mushrooms, but they consume an impressive 67 species from 31 genera (Sawada et al. 2014).

Another notable example is consumption of subterranean fungi by multiple ape species: bonobos (*Pan paniscus*) consume truffle species *Elaphomyces labyrinthinus* and *Hysterangium bonobo* (Bermejo et al. 1994; Elliott et al. 2020; Lucchesi et al. 2021; K. Nara & T. Furuichi, personal communication in Lucchesi et al. 2021). Nutritional analyses of the truffle species *Hysterangium bonobo* consumed by bonobos indicate that the fungus provides more sodium than other foods in the diet, suggesting a key micronutrient source, even if consumed less frequently than other foods (Lucchesi et al. 2021). Western lowland gorillas (*Gorilla gorilla gorilla*) were also observed eating the subterranean fungus *Elaphomyces labyrinthinus*: truffle foraging was observed on 16% of observation days, with variation across four gorilla groups (0.8%–41.7% of observation days) (Abea et al. 2024).

In some cases of primate mycophagy, researchers suggest seasonal variation in mushroom consumption (Porter 2001; Porter and Garber 2010; Schulze et al. 2025). For example, in Issa Valley, Tanzania, three sympatric primate species (*Pan troglodytes schweinfurthii*, *Cercopithecus ascanius*, and *Papio cynocephalus*) consumed mushrooms more during the wet season, when mushrooms were abundant, though one species (*P. cynocephalus*) incorporated mushrooms into the diet even in periods of low mushroom abundance (Schulze et al. 2025). Over ten years of behavioral data on multiple groups of Goeldi's monkeys in Bolivia, the monkeys exhibited seasonal variation in mushroom consumption, relying more on fungi and plant exudate compared to fruit during the dry season, when fruits were less available (Porter and Garber 2010).

Members of multiple fungal genera that humans enjoy have been observed consumed by other primates at some sites. For example, in Issa Valley in Tanzania, humans and nonhuman primates eat chanterelles (*Cantharellus platyphyllus* consumed by humans and chimpanzees [*Pan troglodytes*]*; C. symoensii* consumed by humans and yellow baboons [*Papio cynocephalus*]), milk caps (*Lactifluus rubiginosus* consumed by humans and yellow baboons; *L. edulis* consumed by humans, baboons and chimpanzees) and termite mushrooms (*Termitomyces* cf. *aurantiacus* and *T*. cf. *titanicus* consumed by humans and chimpanzees) (Schulze et al. 2025). *Oudemansiella canarii* (porcelain fungus) is consumed by people (Ruegger et al. 2001; Alberti et al. 2021) and by spider monkeys (*Ateles chamek*, Duerr 2023).

Multiple species of baboons have been recorded eating mushrooms (*Papio anubis*: Rowell 1966; Okecha and Newton‐Fisher 2006; *P. cynocephalus*: Post 1982; Altmann 1998, 2009; Schulze et al. 2025; *P. ursinus*: Byrne et al. 1993; Katsvanga et al. 2009; Johnson et al. 2013; Pebsworth 2020), though, as with many other primates, many descriptions of mycophagy lack detail. For example, sometimes otherwise detailed accounts of the baboon diet include mushrooms in the food category “other” or as “mushroom spp.” Some baboon studies identify fungal foods to the genus and very rarely to the species level. Among Altmann's (1998, 2009) detailed accounts of young yellow baboon diet and nutrition, he observed a yearling baboon eat “the fruit caps of a white mushroom (*Agaricus* prob. *bukavuensis*)” (Altmann 2009, 621). Likewise, Byrne and colleagues (1993) observed adult male and juvenile individuals in one group of chacma baboons in the Drakensberg mountains of South Africa consume “field mushrooms,” implying genus *Agaricus*, which is commonly found in the surrounding area. Yellow baboons in Issa Valley, Tanzania, have been observed consuming chanterelles (*Cantharellus symoensii*), milk caps (*Lactifluus rubiginosus*, *L.s* cf. *heimii*, *L*. cf. *pelliculatus*, *L. edulis*), and brittlegills (*Russula cellulata*, *R*. cf. *phaeocephala*) (Schulze et al. 2025). Additionally, compared to sympatric chimpanzees and red‐tailed monkeys, the yellow baboons at Issa consumed fungi throughout the year: baboon mushroom consumption peaked (36% of baboon feeding observations) in January (the middle of the wet season) but persisted at lower levels throughout the year, with fungal consumption by baboons averaging 11% of baboon feeding observations over the 4 years of the study (Schulze et al. 2025).

Mushroom eating has been documented in other populations of chacma baboons, but researchers rarely identify mushroom species eaten (but see Pebsworth 2020). In Tokai Forest, Western Cape, South Africa, 15% of one adult female chacma baboon's naturally derived (as opposed to human derived) diet consisted of “mushrooms” (Johnson et al. 2013). Chacma baboons in Wildcliff Nature Reserve in Western Cape, South Africa, were observed consuming the sporing bodies of one mushroom species, *Boletus edulis*—commonly known as porcinis, which are highly prized culinary mushrooms to humans—and this identified mushroom's consumption was grouped with other “minor dietary items” during the 2 year study (Pebsworth 2020). Our study reports multiple mushrooms and other fungi, identified to the species level, observed consumed by chacma baboons in Nature's Valley, South Africa. We then assess overlap with other reported fungal species eaten by primates and potential nutritional and other health implications of consumption of these fungi based on human and other mammalian mycophagy literature.

## 2 Methods

### 2.1 Ethical Statement

The wildlife research protocol reported in this manuscript, which involves non‐invasive observation of wild primates, was approved by South African National Parks and by the Institutional Animal Care and Use Committees of both the University of Wisconsin‐Madison and Harvard University. The protocol also adheres to the American Society of Primatologists Ethical Principles for the Treatment of Nonhuman Primates and to the joint International Primatological Society and American Society of Primatologists Code of Best Practices for Field Primatology (http://www.asp.org/resources/docs/Code%20of_Best_Practices%20Oct%202014.pdf).

### 2.2 Study Site and Subjects

The group of chacma baboons in this study range (home range expanded during the study period, 4.83–6.12 km^2^) in the Tsitsikamma Protected Area of the Western Cape of South Africa, in a mosaic of Afrotemperate forest, fynbos, pine plantation, and residential spaces including the town of Nature's Valley (Lubke 2010). The study area is in the eastern Cape Floristic Region, which experiences rainfall year‐round (unlike the western Cape Floristic Region's seasonal winter rainfall; Rebelo et al. 2006; Mucina et al. 2006), while also experiencing temperature fluctuations in distinct autumn, winter, spring and summer seasons (Mucina et al. 2006). Temporal changes in fungal abundance at the site are not currently well characterized and, given the year‐round rainfall, likely do not follow a typical wet season abundance pattern. During the study period of August 2023 to July 2025, the baboon study group consisted of 8–21 individuals including 5–9 adult females, 0–1 adult males, and 3–11 immature individuals, including four infants born over the course of the study. All baboons were habituated and individually identifiable. Although no adult males were observed in the group during August 2023–December 2024, the social peculiarities of the group are unlikely to be relevant to or have an impact on the nature of mushroom‐eating.

### 2.3 Behavioral Data Collection

Observations of consumption of mushrooms and other fungi were recorded during the Nature's Valley Baboon Project's behavioral data collection protocol and on an *ad libitum* basis. The project's behavioral protocol includes 10‐min focal follows collected on all weaned members of the baboon group that include records of feeding and social behaviors. At the group‐level, observations of feeding, social behavior, and group movement are recorded on an all‐occurrence basis. Additionally, at 10‐min intervals, data are collected on group activity (based on what the majority of individuals in the troop are doing), GPS location of the troop, terrain type, and group spread.

### 2.4 Mushroom Identification

**Figure 1:**
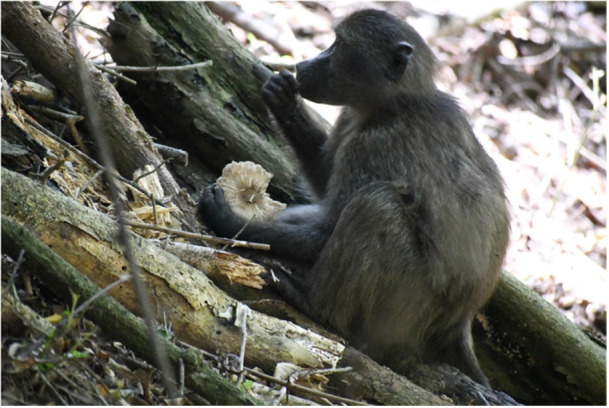
Juvenile female chacma baboon eating *Pluteus cervinus* (deer mushroom) in Nature's Valley, South Africa. Photograph by Nature's Valley Baboon Project.

**Figure 2:**
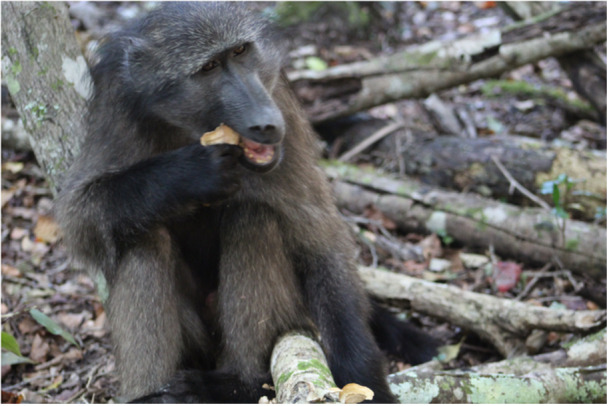
Juvenile male chacma baboon eating *Stereum ostrea* (false turkey‐tail) in Nature's Valley, South Africa. Photograph by Nature's Valley Baboon Project.

**Figure 3:**
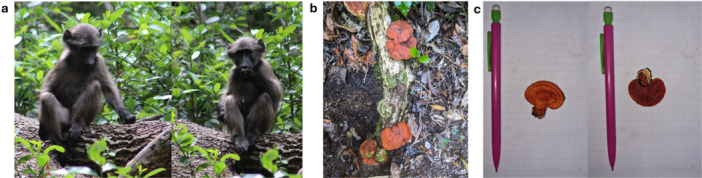
Depicts the process from observation to identification. (a) A young juvenile baboon in Nature's Valley, South Africa, plucking and eating fire bracket fungi (*Pycnoporus sanguineus*). (b) An *in situ* image of the fungus that was eaten. While the juvenile picked all of the available sporing bodies on the log that they ate from, there were additional mushrooms growing nearby. (c) Type images of the top and underside of the sporing body showing the characteristics of the cap and the pore surface.

When mushroom‐eating was observed, our team took multiple steps to support identifying the mushroom to the species level. During the feeding bout, we recorded what part(s) of the mushroom was consumed, whether any parts were spit out instead of being swallowed, and any notable details about the fungal body itself, including texture and whether biting the mushroom produced staining. Whenever possible, we photographed or video recorded the mushroom feeding bout (Figures 1, 2 and 3a). After mushroom consumption ended, we took photographs of any remaining mushrooms or mushroom parts that could aid in species identification. Whenever possible, we took the following series of photographs for each mushroom feeding bout: the mushroom *in situ* in its growth medium or substrate from multiple angles to identify what it was growing from and characterize the growing conditions (Figure 3b); individual photographs to catalog any additional identifying features of the mushroom including both the whole fungal body as well as its parts from multiple angles. In addition to *in situ* photographs, specimen photographs were taken on a white background using either a pencil or a field notebook with a rule as a scale (Figure 3c). During sample collection, we recorded other notable identifying features of a fungal sample, including odors, staining or exuded latex when we scratched or cut the sample, and textures and colors of each part of the fungal body. Whenever the fungal body included a mushroom cap and one was left over after the baboons finished eating, we collected samples of the cap to create a spore print (Storey 2005). Both MAHB and KHS have received training from academic and mycology societies to identify mushrooms, including many of the species in this study. We used all available evidence in consultation with mushroom identification field guides (i.e., Goldman and Gryzenhout 2019) and international and local experts to identify each fungus consumed by members of the baboon group to the species level. Though samples were collected for identification purposes, no voucher specimens were collected for accession to a local herbarium for the species observed because the fungal species were well‐known macrofungi in the area confirmed by both amateur local foragers and experts.

## 3 Results

During the study period, we observed 19 baboons eat fungi at least once (100% of the total unique weaned individuals in the group during the study period), and they fed on 13 different identified fungi (3 identified to the genus level, 10 identified to species level, Table 1; images of all consumed species are in Supplementary Figure 1). During the study period, we also observed the baboons feeding on plant parts (young leaves, mature leaves, flowers, unripe fruits, ripe fruits, roots, underground storage organs, seeds) from 88 identified plant species, in addition to insects (including termites, ants, and aphid larvae), and soil. The baboons were observed feeding on 134 identified unique food items during the study period. Identified (to genus or species level) fungi made up 10% of identified food items.

**Table 1:** For each identified species of fungus eaten by the chacma baboons in Nature's Valley, South Africa, during the study period, in order of observation: fungal species scientific name, common name and part(s) consumed; number of baboons and age and sex classes of those baboons who consumed the fungal species; and information about the month, season and time of day the species was consumed.

Fungus			Baboons consuming fungus		Time of consumption		
Scientific name	Common name	Part(s) consumed	Number of unique individuals	Age/sex classes	Month(s)	Season(s)	Time(s) of day
*Pluteus cervinus*	Deer mushroom	Cap	2	JF, JM	Nov, Dec	Summer	Afternoon
*Stereum ostrea*	False turkey‐tail	Sporing body	9	AF, JF, JM	Aug, Sept, Oct, June	Winter, spring	Morning, afternoon, late afternoon
*Hymenopellis radicata*	Rooting shank	Cap + stipe	1	JM	Oct	Spring	Late afternoon
*Pycnoporus sanguineus*	Fire bracket	Sporing body	1	JM	Nov	Spring	Afternoon
*Phaeotremella foliacea*	Jelly fungus	Sporing body	1	AF	Oct	Spring	Afternoon
*Hohenbuehelia petaloides*	Shoehorn oyster mushroom	Sporing body	1	AF	April	Autumn	Afternoon
*Trametes cingulata*	Black cork polypore	Sporing body	1	JM	April	Autumn	Afternoon
*Russula capensis*	Cape Russula	Cap + stipe	5	AF, JM	May	Autumn	Morning
*Schizophyllum commune*	Splitgill mushroom	Sporing body	2	AF, JF	June	Winter	Afternoon
*Boletus edulis*	Porcini or King Bolete	Varied	18	AF, AM, SM, JM, JF	July	Winter	Afternoon

Abbreviations for Age/Sex classes: AF, adult female; AM, adult male; JF, juvenile female; JM, juvenile male; SM, subadult male.

In December 2023, a juvenile female baboon fed on the caps of fully mature deer mushrooms, *Pluteus cervinus* (Figure 1), and almost 1 year later, an older juvenile male was observed eating caps of *P. cervinus* in November 2024.

Baboons fed on the sporing bodies of *Stereum ostrea*, commonly known as false turkey‐tail (Table 1; Figure 2): In August 2024, eight individuals (adult females = 5, one of which was nulliparous; juvenile females = 1; juvenile males = 2; 89% of the study group members at that time) fed on *S. ostrea*. In September 2024, one juvenile female and one adult female (both of which also fed on *S. ostrea* in August) fed on the fungus again. In June 2025, one adult female who joined the group several months prior to the observation, fed on *S. ostrea* (at which point she was one of 19 group members).

In October 2024, an older juvenile male ate the cap and stipe of *Hymenopellis radicata* (rooting shank; Table 1). In November 2024, a young juvenile was observed eating whole *Pycnoporus sanguineus* (fire bracket or cinnabar polypore; Table 1; Figure 3). A nulliparous adult female was observed eating the whole sporing body of *Phaeotremella foliacea* (jelly fungus; Table 1) in October 2024, and a second nulliparous female was observed eating the whole sporing body of *Hohenbuehelia petaloides* (shoehorn oyster mushroom; Table 1) in April 2025. A juvenile male was observed eating black cork polypore (*Trametes cingulata*; Table 1) in April 2025. In May 2025, multiple individuals, including three parous adult females, one nulliparous female, and one juvenile male, consumed the caps and stipes of Cape Russula (*Russula capensis*; Table 1) while foraging in a pine plantation. In June 2025, a parous adult female and her young juvenile female both fed on *Schizophyllum commune* (splitgill mushroom; Table 1); the young juvenile was observed feeding on *S. commune* after the mother.

In July 2025, all members of the baboon group at that time excluding neonates (*n* = 18), including the adult male, subadult male, adult females, and juveniles, consumed *Boletus edulis* (porcini or King Bolete; Table 1) in a pine plantation continuously for over 1 h. Multiple individuals, including a juvenile male, stuffed their cheek pouches with *B. edulis* while continuing to forage on *B. edulis* by shifting and digging in the pine needle duff with their hands. The component of the fungus (cap, stipe, whole) eaten varied across individuals and for a given individual over the feeding period.

## 4 Discussion

Over 23 months of observing a habituated group of free‐ranging chacma baboons in Nature's Valley, South Africa, we recorded baboons eating 13 fungi, 10 of which we identified to the species level (Table 1). In a habitat that experiences four seasons and year‐round rainfall (Mucina et al. 2006), we observed fungi consumption across all seasons. Identifying these fungal species consumed is an important initial step to understanding the importance and potential uses of fungi in baboon diets, as well as contextualizing the ecosystem roles of baboons as potential spore dispersers.

Several fungal species consumed by the baboons in Nature's Valley are used by humans in culinary and medicinal contexts (Table 2). The most prominent example is *Boletus edulis*, commonly referred to as porcini or King Bolete, which is highly prized for its flavor and texture across the globe, especially in North America, Europe and Asia. Although this species is not indigenous to South Africa, it grows in association with pine plantations from European stock (Marais and Kotze 1977) and it is also consumed by members of at least one other population of chacma baboons that also has pine plantations in their home range (Pebsworth 2020). *Schizophyllum commune* (consumed by the baboons in this study as well as by Japanese macaques on Yakushima Island, Sawada et al. 2014) is also eaten by humans all over the world (Asia: Singh et al. 2015; Abd Razak et al. 2024; Singh et al. 2021; Central America: Ruan‐Soto et al. 2025; Africa: Milenge Kamalebo et al. 2018).

**Table 2:** For each identified species of fungus eaten by chacma baboons in Nature's Valley, South Africa, during the study period: species scientific name, species common name, fungal functional group, other nonhuman primates who have been reported to consume it, reported human culinary and medicinal uses, and references.

Species	Common names	Fungal functional group	Other primate species	Human culinary use	Human medicinal use	References
*Boletus edulis*	King Bolete, porcini	Mycorrhizal	*Papio ursinus* eat *Boletus edulis* (at Wildcliff Nature Reserve in Western Cape, South Africa)	Edible, one of the most highly prized culinary mushrooms worldwide	Potential medicinal uses (multiple), but not traditional	Pebsworth (2020); Tan et al. (2022); Vamanu and Nita (2013); Vidović et al. (2010)
*Hohenbuehelia petaloides*	Shoehorn oyster mushroom	Decomposer	*Macaca fuscata yakui* eat *Hohenbuehelia* sp.	Edible, but widely considered unpalatable	Potential medicinal uses (antimicrobial properties), but not traditional	Sawada et al. (2014) appendix; Korkmaz et al. (2024)
*Hymenopellis radicata*	Rooting shank	Decomposer		Edible, but widely considered unpalatable	Potential medicinal uses (anti‐microbial, anti‐viral properties), but not traditional	Niego et al. (2021)
*Phaeotremella foliacea*	Jelly fungus, jelly brain	Mycoparasitic^a^		Edible, but not prized	No known medicinal uses	Spirin et al. (2018)
*Pluteus cervinus*	Deer mushroom	Decomposer	*Macaca fuscata yakui* eat *Pluteus granularis*	Edible, but not prized	No known medicinal uses	Sawada et al. 2014 appendix
*Pycnoporus sanguineus*	Cinnabar polypore	Decomposer		Inedible	Traditionally used to treat infection and fever in multiple cultures (anti‐inflammatory properties)	Borderes et al. (2011); Haro‐Luna et al. (2019); Jouda et al. (2018); Kalotas (1996); Li et al. (2020); Milenge Kamalebo et al. (2018)
*Russula capensis* b	Cape Russula; Cape brittlegill	Mycorrhizal	*Macaca fuscata yakui* eat 18 *Russula* spp.; *Presbytis rubicunda* eat 2 *Russula* spp.; *Papio cynocephalus* eat *Russula cellulata*, *Russula* cf. *phaeocephala*	Edible (choice), eaten locally in South Africa and similar regional varieties are eaten in other countries	Potential medicinal uses (multiple), but not traditional	Sawada et al. (2014) appendix; Cheyne et al. (2019); Schulze et al. (2025)
*Schizophyllum commune*	Splitgill mushroom	Decomposer, though sometimes parasitic^c^	*Macaca fuscata yakui* eat *Schizophyllum commune*	Edible, but not prized	Widely used as a traditional medicine (multiple medicinal properties)	Sawada et al. (2014) appendix; Abd Razak et al. (2024); Albina et al. (2023); dela Cruz and De Leon (2023); Kumar and Xu (2025); Oyetayo (2011)
*Stereum ostrea*	False‐turkey tail	Decomposer	*Macaca fuscata yakui* eat *Stereum ostrea*	Inedible	Traditionally used to treat general inflammation and respiratory symptoms (antimicrobial and antioxidant properties)	Sawada et al. (2014) appendix; Imtiaj et al. 2007; Praveen et al. (2012)
*Trametes cingulata*	Black cork polypore	Decomposer		Inedible	Potential medicinal uses (antimicrobial properties)	Ofodile et al. (2008)

a This fungus is not parasitic in the common sense of fungal functional guilds (with plants) but parasitic to mycelium of other fungi, and therefore labeled mycoparasitic (Spirin et al. 2018).

^b^ Older *Russula* spp. specimens are known to cause gastrointestinal distress in humans due to presence of a *Mycogone* species of mold (Goldman and Gryzenhout 2019).

c This is a versatile fungus that can act as a decomposer, tree wound parasite, and pathogen to plants (Kleijburg and Wösten 2025).

While the Cape Russula (*Russula capensis*) is endemic to South Africa and is eaten by local people in South Africa, similar local varieties of *Russula* are prized in Scandinavian and Eastern European cuisine (Dugan 2011). Interestingly, older specimens of this species can induce feelings of inebriation due to infection by *Mycogone* fungus known to attack this species (Goldman and Gryzenhout 2019); no baboons in the study group were observed eating older specimens. Researchers have documented several primate species consuming *Russula* mushrooms, including yellow baboons in Tanzania (*R. cellulata*, Schulze et al. 2025), Japanese macaques on Yakushima Island (Sawada et al. 2014), and red langurs (*Presbytis rubicunda*) in Borneo (Cheyne et al. 2019).

Two other species consumed by the baboons in this study, *Phaeotremella foliacea* and *Hohenbuehelia petaloide*, are considered less appealing in a culinary context to humans and have a tougher texture compared to other edible fungi, but both are dried and used in soups and stews, particularly in East Asia (Spirin et al. 2018). In addition to culinary uses, a recent pharmacological study on *Hohenbuehelia petaloide* revealed compounds with anti‐inflammatory and antimicrobial properties (Korkmaz et al. 2024). Interestingly, despite widespread distribution, this species has not been traditionally used as a medicinal mushroom.

Other fungi eaten by the baboons do feature in traditional medicine. For instance, *Schizophyllum commune* has been regarded as a “health food” generally and used to treat diabetes in Nigeria (Oyetayo 2011) and is common in traditional diets of several ethnic groups in Democratic Republic of Congo where it is also used medicinally to treat wounds and breast inflammation (Milenge Kamalebo et al. 2018). Several studies have confirmed its potential in treating chronic ailments, including diabetes (Kumar and Xu 2025). *Pycnoporus sanguineus* has been used in Jalisco, Mexico, to treat gastrointestinal symptoms and kidney dysfunction as well as fever more generally (Haro‐Luna et al. 2019), and *P. sanguineus* (and *P. coccineus*) are used by Aboriginal Australians in treatment of dental and skin ailments through a mild numbing effect (Kalotas 1996). As with *S. commune*, a pharmacological study confirmed the clinical potential of a polysaccharide derived from *P. sanguineus* for treating irritable bowel syndrome by relieving colonic lesions and restoring intestinal homeostasis (Li et al. 2020). Determining the potential health value of these fungal species to baboons and other primates will require not only increasing our attention to identifying the species that are targeted by wild primates, but also collecting and analyzing samples of previously untested fungal species.

What nutritional value can fungi provide to primates? Wild and cultivated mushrooms in the human diet are often touted as key protein sources, especially for vegetarian diets (Valverde et al. 2015; Pashaei et al. 2024). Some species of wild mushrooms eaten by humans in Mexico were found to contain high levels of protein (*Amanita rubescens* = 17.4%; *Exsudoporus frostii* [formerly *Boletus frostii*] = 15.8%; *Lactarius indigo* = 14.5%; *Ramaria flava* = 13.4%,) and to be important sources of essential amino acids like lysine, threonine and phenylalanine (León‐Guzmán et al. 1997). Similarly, wild mushrooms used in culinary and medicinal contexts in Tanzania demonstrated that diverse mushroom species (*Boletus pruinatus* or *Xerocomellus pruinatus* [common name: matte bolete]; *Cantharellus cibarius* [golden chanterelle]; *Pleurotus sajor‐caju* or *Lentinus sajor‐caju* [funnel woodcap]; *Russula hiemisilvae* [brittlegill]; *Boletinus cavipes* or *Suillus cavipes* [hollow‐foot bolete]; and *Ganoderma lucidum* [reishi or lacquered bracket]) contain multiple essential amino acids (Mdachi et al. 2004).

Because it is so widely eaten by humans across the world, *Boletus edulis* – a species observed consumed by members of at least two populations of chacma baboons (this study; Pebsworth 2020) – has been analyzed for macronutrient (and micronutrient, see below) content (Heleno et al. 2011; Falandysz 2021; Tan et al. 2022). Different parts of *B. edulis* have different nutritional value: one study found that the caps of raw *B. edulis* had higher concentrations of protein and fat compared to the stipes, which had higher carbohydrate content, though mostly in the form of structural carbohydrates (Mena García et al. 2021). Therefore, the macronutrient, especially protein, gains from some fungi consumed by chacma baboons are considerable, especially in the context of long foraging bouts like the observations of multiple chacma baboons in this study consuming *B. edulis* caps, stipes and whole mushrooms for over 1 h.

Studies analyzing macronutrient composition of mushrooms and other fungi consumed by nonhuman primates are rare, with the exception of callitrichid mycophagy nutrition (Hanson et al. 2006; Hilário and Ferrari 2011). The sporocarps of *Mycocitrus* species of fungi eaten by buffy‐headed marmosets (*Callithrix flaviceps*) (Hilário and Ferrari 2011) and of *Auricularia* spp. and *Ascopolyporus* spp. eaten by Goeldi's monkeys (*Callimico goeldii*) (Hanson et al. 2006) were low in fat and protein and high in carbohydrates (though these are composed of carbohydrates of varying digestibility, including easy to access sugar and less‐ or non‐digestible chitin). These callitrichid mycophagy nutrition studies also point to the variation in protein gains that diverse fungi provide to primates, as the fungi consumed by these monkeys were low in protein content.

Digestibility of mushrooms and resulting availability of nutritional components to the consumer vary by characteristics of the mushroom and by digestive physiology of the consumer. Variation in digestive physiology across primates likely leads to variable nutritional value of mushrooms, with some primates able to extract nutrients and energy from fungi through microbial activity in complex guts, similarly to foregut fermenting marsupials (Claridge and Cork 1994; McIlwee and Johnson 1998). Foregut fermenting primates that consume fungi (e.g., langurs, de Groot and Nekaris 2016; Cheyne et al. 2019; colobus monkeys [species not specified, either *Piliocolobus rufomitratus* or *Colobus guereza*] eating *Pleurotus* sp., K. Hodge, pers. obs. in Hanson et al. 2003; snub‐nosed monkeys eating *Pleurotus* spp., Zhang et al. 2024) may experience improved mushroom nutritional gains compared to primates with simpler guts. Variable levels of chitin, the polysaccharide of fungal cell walls, in mushrooms (Vetter and Siller 1991) may make digestion of mushrooms more challenging for some primates compared to others given differences in digestive physiology and chitinase presence or absence from the gut (Janiak et al. 2018).

Availability of nutrients from fungi to primates also varies by preparation: in contrast to nonhuman primates, humans rarely eat raw fresh mushrooms and frequently prepare fresh fungi for consumption via cooking methods which affect aspects of chemical composition. *Boletus edulis* caps and stipes were reduced in protein content by grilling or roasting (Mena García et al. 2021), with implications for lower protein gains by humans eating cooked *B. edulis* versus nonhuman primates eating raw fresh *B. edulis*. Cooking has been found to affect chitin content of some mushrooms (fresh cultivated *Agaricus bisporus*: Manzi et al. 2001; fresh cultivated *Agaricus bisporus*, *Grifola frondosa* (maitake), and *Lentinus edodes* (shiitake): Dikeman et al. 2005), while for other mushroom species cooking has no significant effect on chitin content (dried wild *Boletus* spp.: Manzi et al. 2004), with potential implications for differences in the levels of chitin the consumer has to contend with for human versus nonhuman primates. As cooking mushrooms reduces levels of certain toxic compounds and elements (Chiocchetti et al. 2020; Vodovar et al. 2024), this may indicate that nonhuman primates must engage in more physiological detoxification or face higher risk of poisoning than humans when consuming some raw fungi. There is ambiguity in whether toxicity always corresponds similarly in nonhuman primates, as some fungi indicated as toxic to humans are consumed by nonhuman primates (Sawada et al. 2014; Elliott et al. 2022).

Fungi are excellent at accumulating elements from their environment, which can lead to “nutritional asset” or “toxicological risk” (Kalač 2010, 2). In terms of nutritional asset, in addition to macronutrients, fungi also provide micronutrients, including minerals, to mycophagous primates. Humans consume fungi with diverse composition of minerals (Agrahar‐Murugkar and Subbulakshmi 2005; Kalač 2009; Nakalembe et al. 2015; Haro et al. 2020). *Boletus edulis* found in pine plantations in KwaZulu‐Natal, South Africa, were good sources of potassium (linked to the symbiotic relationship with pine trees which require potassium and phosphorous) as well as zinc, copper and selenium (Rasalanavho et la. 2020). For nonhuman primate micronutrient nutrition of mycophagy, multiple essential minerals were found in fungi (*Auricularia* spp. and *Ascopolyporus* spp.) eaten by Goeldi's monkeys (*C. goeldii*; Hanson et al. 2006), with the fungi highest in potassium compared to other minerals (Hanson et al. 2006). The case of the high sodium content of the *Hysterangium bonobo* truffle compared to other foods eaten by bonobos (Lucchesi et al. 2021) points to the crucial micronutritional value of some foods regardless of how often they are incorporated into the diet (*sensu* decaying wood rarely consumed by mountain gorillas but a key source of sodium in the diet, Rothman et al. 2006). Sodium is a mineral known to be limiting in tropical environments (Venable et al. 2020), so foods—in the bonobo case a fungus—that can provide this mineral that is rare in many primate habitats, are crucial for wild primate health. The extent to which mushrooms and other fungi provide certain micronutrients more than other foods available to nonhuman primates likely varies widely by fungal species and availability of micronutrients in a given environment (e.g., Afrotemperate forests and Afrotropical forests have different soils and different limiting minerals). In terms of toxicological risk, mushrooms readily absorb metals from substrates, so they can contain high levels of heavy metals in more polluted anthropogenic environments (Kalač 2000), with implications for human health (Bucurica et al. 2024). Another unexplored area of anthropogenic change and primate mycophagy is the potential consumption of heavy metals. Therefore, the consumption of mushrooms by nonhuman primates in anthropogenic landscapes may also lead to consumption of compounds with potential detrimental health effects.

Mushrooms and other fungi also can provide consumers with other micronutrients like vitamins, some of which are antioxidants (Agrahar‐Murugkar and Subbulakshmi 2005; Jaworska et al. 2015; Nakalembe et al. 2015; Cardwell et al. 2018). A mushroom species eaten by baboons in this study and by humans, *Boletus edulis*, has been found to contain vitamin C and tocopherols (Heleno et al. 2011). Antioxidant activity for *B. edulis* and other mushrooms are altered by cooking (Jaworska et al. 2015; Mena García et al. 2021) with implications for differences in what wild primates gain from raw fungi versus what humans gain from cooked fungi. Future work is needed on wild primate vitamin nutrition, including antioxidants, in the context of mycophagy.

The high water content of many mushroom sporing bodies also has hydration purpose implications for mammalian mycophagy (Luoma et al. 2003), especially in seasonal habitats and habitats impacted by climate change. That said, water content is variable across species of fungi and developmental stages of fungi. For example, Hanson et al. (2006) found that the water content of the mushrooms consumed by Goeldi's monkeys varied between 55% and 88% (moisture content of one sample of *Ascopolyporus polychrous* = 70%; four samples of *Ascopolyporus polyporoides* = 55% ± 3.2%; one sample of *Auricularia auricula* = 88%; five samples of *Auricularia delicata* = 88% ± 2.9%); it is important to note that the moisture variation for mushrooms consumed by Goeldi's monkeys is reflective of some of the species analyzed, with jelly fungi (*Auricularia* spp.) exhibiting extreme variation in moisture linked to their life cycle and adaptability. In addition to consideration of high water content of fungi for hydration, low water content leading to dry and textured fungi may serve functions via physical properties, similarly to some leaves swallowed by primates to alleviate intestinal symptoms associated with high parasite loads (Huffman et al. 1997, 2012). The chacma baboons' consumption of the bracket fungus *Stereum ostrea* even when in a tougher and drier state may suggest such medicinal purposes, but further investigation is needed.

Primates, like other mushroom‐eaters such as rodents and marsupials (Frank et al. 2009; Johnson 1995), may serve as spore dispersers, playing a key ecosystem role. Elliott and colleagues' (2022) review reported that experimental studies show that 58 mycorrhizal fungi remain viable after gut passage through mammals. Some studies suggest that the digestive process facilitates spore germination (Maser et al. 1978; Piattoni et al. 2014; Ori et al. 2018, 2021). Studies on animal‐fungal relationships point to mammalian fungal dispersers being crucial to increasing fungal genetic diversity (Fogel and Trappe 1978; Gehring et al. 2002; Nuske et al. 2017; Elliott et al. 2022), as well as to tree succession (Ashkannejhad and Horton 2006; Stephens et al. 2021). Defecation of still‐viable spores has yet to be demonstrated in nonhuman primates. The consumption by the baboons in this study of ten species of mushrooms (in addition to three more fungi identified to the genus level) points to diverse fungal consumption in an Afrotemperate forest and suggests a need to better understand the potential for spore dispersal by baboons. The Nature's Valley baboon group has a smaller home range than is typical of chacma baboons, even after an expanded home range, but given a home range of 4–6 km^2^ and an estimated gut passage time of 37 h (based on experimental marker studies with *Papio cynocephalus*, Clemens and Phillips 1980), their hypothetical potential for spore dispersal is larger than smaller bodied mammals at the site. In future work, we will model spore dispersal potential by the baboons (*sensu* Danks et al. 2020; Stephens et al. 2025).

As demonstrated by chacma baboon consumption of *Boletus edulis* (this study; Pebsworth 2020), human introduction of exotic fungi has implications for nonhuman primate diet and primate habitats. Though the incorporation of introduced plants in the primate diet has been explored across taxa (Bicca‐Marques and Calegaro‐Marques 1994; Noer et al. 2025; Wimberger et al. 2017) including in baboons (Paterson 2006; Pamla et al. 2024; Pebsworth 2020), the consumption of introduced fungi has rarely been examined. The consumption of *B. edulis*, a fungus native to North America, Europe and Asia, by chacma baboons in South Africa at multiple sites points to the need for exploration of exotic as well as indigenous fungi in primate habitats. Just as indigenous and exotic plant species contribute to primate diet and health, as well as potentially to helping or hindering maintenance or reforestation of indigenous forests, somewhat similar dynamics may be true with mushrooms in anthropogenic and mosaic landscapes.

## 5 Conclusion & Future Directions

As primate mycophagy is both relatively rare and understudied, it is unclear exactly what baboons and other primates are getting from these foods, and it likely varies by fungal species, primate digestive physiology, and additional factors. However, regardless of how often mushrooms and other fungi are consumed by any species, they still hold potential primate health implications. Given the diversity of mushroom species and the resulting diverse nutritional (Wallis et al. 2012; Wasser 2014) and medicinal (Yamin‐Pasternak 2011; Wasser 2014) composition of mushrooms indicated for human primates, fungi can potentially play diverse roles in nonhuman primate ecology.

Nonprimate mammalian mycophagy studies rely heavily on microscopic fecal analyses (Elliott et al. 2022). Microscopic methods of fungal identification involve multiple approaches to mounting animal fecal material on a slide and examining it to at least 400x magnification to identify fungi based on spore morphologies, usually to the family or genus level (Gordon and Comport 1998). Such analyses allow researchers to infer fungal ingestion via observation of a high volume of spores as well as dispersal potential based on intact spores in the sample which indicates viability in multiple mammals (Elliott et al. 2022). One challenge to identifying fungi from spores is that comprehensive fungal inventories (i.e., surveys and documentation of fungal diversity) of an area from which animal fecal samples were collected may not be available; however, regional fungal keys (i.e., systematic, often dichotomous, fungal identification tools) are often available (Mueller et al. 2011; Elliott et al. 2022). Microscopic analyses can be well complemented by molecular analyses (e.g., Borgmann‐Winter et al. 2023), which enable identification of fungal operational taxonomic units (OTUs) in fecal samples. However, molecular results should be interpreted with caution as they may include fungi intentionally consumed by the animal, fungi or spores consumed incidentally along with other food items, fungi originating in the animal's gastrointestinal tract, and fungi that colonize or spores that land on the feces after defecation but before collection (Hopkins et al. 2021). Future work, including our own at Nature's Valley, should incorporate fecal analyses, beginning with microscopic methods, which are standard protocol by mycologists and low‐cost.

Examining mycophagy across sites and species is made easier with fungal genera or species level identification. That said, interesting questions also arise about mushroom functional groups. For example, based on molecular analyses of feces, the omnivorous Australian marsupial, the quenda (*Isoodon fusciventer*), fed on mycorrhizal (31% of common OTUs in scat) and saprotrophic (also known as decomposer, 19%) fungi (Hopkins et al. 2021) and mycorrhizal fungal spores were viable after defecation and successfully colonized plant roots (Tay et al. 2018). It remains to be explored if there are certain functional groups of fungi that serve certain (nutritional, health) purposes for primate species and their habitats (see Table 2 for functional groups of fungi in our study).

Identifying and analyzing mushroom species that are consumed by primates, including baboons, is critical to understanding understudied primate‐fungi relationships, with implications for macronutrient, micronutrient, and medicinal gains for primates, as well as primate ecosystem roles. Examining bioavailability of nutritional components in fungi to wild primates will also be key to understanding primate‐fungal relationships and what primates gain from diverse fungi.

## Author Contributions

**Margaret A. H. Bryer:** conceptualization, investigation, funding acquisition, writing – original draft, writing – review and editing, project administration, data curation, supervision, methodology. **Carla van Hasselt:** investigation, writing – review and editing. **Sophie E. Kurilla:** investigation, writing – review and editing. **Sky B. Beveridge:** investigation, writing – review and editing. **Hendri C. Coetzee:** writing – review and editing, supervision, project administration, resources. **Kris H. Sabbi:** conceptualization, methodology, investigation, funding acquisition, writing – original draft, writing – review and editing, data curation, supervision, project administration, resources.

## Conflicts of Interest

The authors declare no conflicts of interest.

## Supporting information


Supporting File 1

## Data Availability

The data that support the findings of this study are available from the corresponding author upon reasonable request.
