# Supplementary material for: Fungal Diversity and Potential Health Benefits of Mycophagy in Chacma Baboons (Papio ursinus)
Source: Am J Primatol. 2026 Apr 12;88(4):e70146. doi: 10.1002/ajp.70146 (PMC13071124; doi:10.1002/ajp.70146)
Supplement: Supplementary file 1 — Supporting File 1 [file AJP-88-e70146-s001.docx]

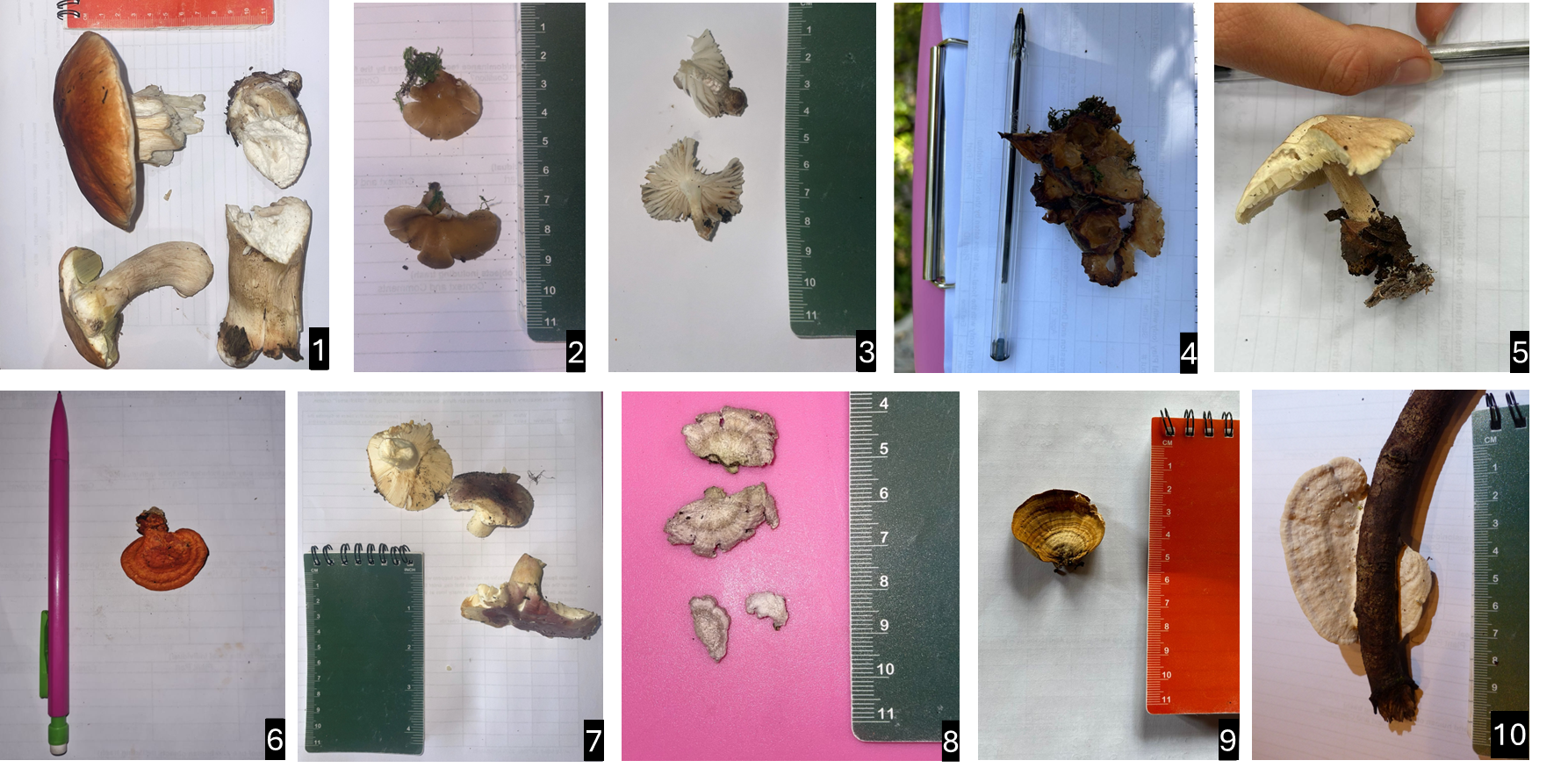


Supplementary Figure 1. Species of fungi observed consumed by chacma baboons in Nature’s Valley, South Africa, in alphabetical order: (1) *Boletus edulis*, (2) *Hohenbuehelia petaloides*, (3) *Hymenopellis radicata*, (4) *Phaeotremella foliacea*, (5) *Pluteus cervinus*, (6) *Pycnoporus sanguineus*, (7) *Russula capensis*, (8) *Schizophyllum commune*, (9) *Stereum ostrea*, and (10) *Trametes cingulata*.
